# Selenium, a Micronutrient That Modulates Cardiovascular Health via Redox Enzymology

**DOI:** 10.3390/nu13093238

**Published:** 2021-09-17

**Authors:** Diane E. Handy, Jacob Joseph, Joseph Loscalzo

**Affiliations:** 1 Cardiovascular Division, Department of Medicine, Brigham and Women’s Hospital and Harvard Medical School, Boston, MA 02115, USA; jjoseph16@partners.org (J.J.); jloscalzo@rics.bwh.harvard.edu (J.L.); 2 Department of Medicine, VA Boston Healthcare System, Boston, MA 02115, USA

**Keywords:** selenium, selenoprotein, cardiovascular disease, reactive oxygen species, endothelial dysfunction, vascular smooth muscle cell, cardiac, thrombosis, atherosclerosis, inflammation

## Abstract

Selenium (Se) is a trace nutrient that promotes human health through its incorporation into selenoproteins in the form of the redox-active amino acid selenocysteine (Sec). There are 25 selenoproteins in humans, and many of them play essential roles in the protection against oxidative stress. Selenoproteins, such as glutathione peroxidase and thioredoxin reductase, play an important role in the reduction of hydrogen and lipid hydroperoxides, and regulate the redox status of Cys in proteins. Emerging evidence suggests a role for endoplasmic reticulum selenoproteins, such as selenoproteins K, S, and T, in mediating redox homeostasis, protein modifications, and endoplasmic reticulum stress. Selenoprotein P, which functions as a carrier of Se to tissues, also participates in regulating cellular reactive oxygen species. Cellular reactive oxygen species are essential for regulating cell growth and proliferation, protein folding, and normal mitochondrial function, but their excess causes cell damage and mitochondrial dysfunction, and promotes inflammatory responses. Experimental evidence indicates a role for individual selenoproteins in cardiovascular diseases, primarily by modulating the damaging effects of reactive oxygen species. This review examines the roles that selenoproteins play in regulating vascular and cardiac function in health and disease, highlighting their antioxidant and redox actions in these processes.

## 1. Introduction

Selenium (Se) has been shown to be an essential micronutrient that modulates cardiovascular, immune, metabolic, and thyroid functions via its incorporation into selenoproteins as the amino acid selenocysteine (Sec). Selenium is acquired in the diet from a variety of sources, including plants and animals. In foods, selenium can be found in inorganic forms, such as sodium selenite and sodium selenate, as well as in organic forms, such as selenocysteine, selenomethionine, methylselenocysteine, and γ-glutamyl methylselenocysteine [1,2]. Cellular metabolism of selenium compounds to the common metabolite selenide allows for the biosynthesis of selenocysteine and its incorporation into selenoproteins [3].

There are 25 selenoprotein genes in humans, and 24 in mice. Selenocysteine is unique in that its biosynthesis occurs on its tRNA (tRNA^(Ser)Sec^) that is first aminoacylated with Serine (Ser). Following the enzymatic phosphorylation of the seryl group to form phosphoseryl- tRNA^(Ser)Sec^ [4], a Se group is transferred from selenophosphate to produce a Sec-charged tRNA. The codon for Sec is UGA, which is the opal (or umber) stop codon. The recognition of a UGA for Sec incorporation instead of as a stop codon involves a dedicated translational mechanism: in addition to the tRNA^(Ser)Sec^, the 3′ untranslated region (UTR) of selenoprotein mRNAs contains a stem loop structure (that is called a Sec insertion sequence or SECIS element) which, together with specific SECIS binding proteins, such as SBP2, are essential for translation. In addition, Sec-insertion requires a special elongation factor, EF^Sec^ [5]. New findings have uncovered a unique variation in this canonical mechanism, in which an additional motif in the 3′ UTR, named a SelS 3′ UTR Positive Recoding or SPUR element, is necessary for optimal Sec incorporation into Selenoprotein S [6].

Structurally, Sec is like Cysteine (Cys), with a Se substituting for the S group in Cys. Similar to Cys, Sec is redox-active and it forms the active site of selenoenzymes. The selenol group (Se-H) has a pK_a_ of 5.2; thus, it is more likely to be deprotonated at neutral pH than the thiol group in Cys, which has a pK_a_ of 8.25 [7]. This intrinsic property renders Sec more reactive than Cys, and may contribute to the greater activity in Sec-containing enzymes than comparable Cys-substituted forms. Sec is also less susceptible to overoxidation than Cys, contributing to its usefulness in mediating antioxidant protection [8].

Reactive oxygen species (ROS) are essential to normal cellular function; at a cellular level they are essential for growth factor-mediated signaling, protein folding and other necessary disulfide-bond formation, and normal mitochondrial function [9,10]. A lack of necessary oxidants can lead to reductive stress, a process that may impair cellular responses to growth factors, promote insulin resistance [11], and contribute to protein misfolding-induced cardiomyopathy [12]. Excess ROS, however, can promote cell and tissue damage, causing protein and DNA damage, altering cellular proliferation, promoting inflammatory responses, and, in extreme cases, promoting cell death by apoptosis or ferroptosis. These cellular changes contribute to the development of atherothrombotic and cardiac disease states [13,14,15,16,17]. Selenoproteins have important roles in redox biology, especially selenoproteins with known roles in antioxidant and redox function, such as glutathione peroxidases (GPxs), thioredoxin reductases (Txnrd), and methionine-sulfoxide reductase (MsrB1). Selenoprotein P, Selenoprotein S, and Selenoprotein T also modulate ROS levels. This review examines the roles that selenoproteins play in regulating vascular and cardiac function in health and disease, highlighting their antioxidant and redox actions in these processes.

## 2. Functional Roles of Selenoproteins Involved in Cardiovascular Homeostasis

**Figure 1:**
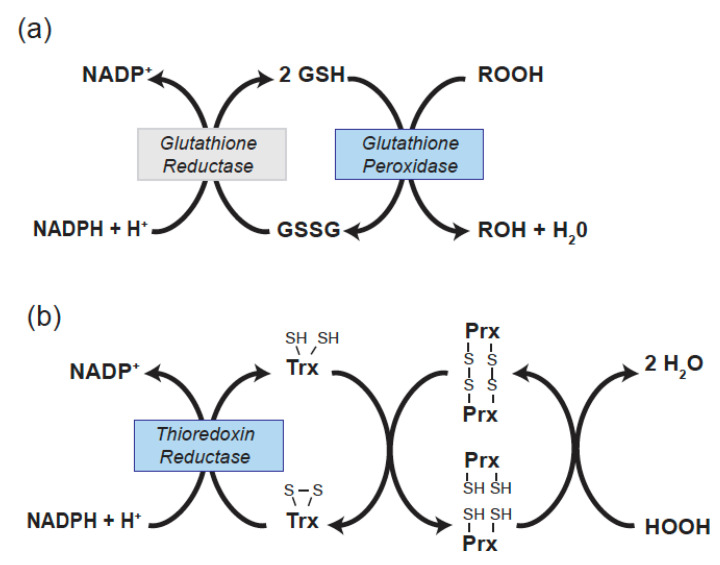
Redox pathways involved in the reduction of cellular peroxides. (**a**) *Glutathione peroxidase* reduces hydrogen and lipid hydroperoxides (ROOH) to the corresponding alcohol (ROH) and water using GSH (*glutathione*) as a reductant, resulting in the formation of oxidized glutathione (GSSG). *Glutathione reductase* is an NADPH-dependent enzyme that maintains the pool of reduced GSH. (**b**) *Thioredoxin reductase* is an NADPH-dependent enzyme that maintains the pool of reduced thioredoxin (Trx). *Thioredoxin* reduces the peroxiredoxin (Prx) proteins that consume hydrogen peroxide. Cys-Cys dithols are indicated by S-S, and reduced thiols by SH in the figure.

There are 5 selenocysteine-containing GPxs in human (GPx1, GPx2, GPx3, GPx4, and GPx6). GPx2 is restricted in its expression to gastrointestinal tissue and GPx6 is restricted to the olfactory system. The remaining Sec-containing GPxs (1, 3, and 4) have been found to have effects on several vascular and cardiac functions. GPx enzymes reduce hydrogen or lipid hydroperoxides using glutathione (GSH) as a reductant. Thus, in reducing peroxides GPxs alter the redox state of GSH, a major cellular thiol, to its oxidized form, GSSG. The NADPH-dependent enzyme glutathione reductase restores GSSG to its reduced form (Figure 1a).

The GPx1 enzyme was the first characterized selenoprotein [18]. It is ubiquitously expressed and is found intracellularly in both the cytoplasm and the mitochondria. GPx3 is expressed primarily in the kidney [19] and is secreted into the blood. Thus, GPx3 was initially called plasma GPx [20,21]. Other tissues also express GPx3 transcripts [22], but it is unclear if expression in other tissues contributes to the circulating concentrations of GPx3. Both GPx1 and GPx3 primarily target hydrogen peroxide and soluble lipid hydroperoxides, they are homotetramers, and in mice their knockout is not lethal. GPx4 is unique in that it is a monomer; it reduces membrane bound lipid peroxides; and it can target to the nucleus, cytoplasm, and mitochondria, although the nuclear form is expressed at very low levels [23,24]. In mice, knockout of GPx4 results in embryonic lethality. In addition, its inhibition can promote a form of cell death called ferroptosis that involves iron-dependent lipid peroxidation [25]. The GPxs have been shown to regulate endothelial and cardiac functions that are involved in cardiovascular diseases.

Molecular cloning methods contributed to the identification of distinct thioredoxin reductases (Txnrds) that are involved in redox-regulation in the cytoplasm (Txnrd1) [26] and mitochondria (Txnrd2) [27]. In mice, lack of either Txnrd1 or Txnrd2 causes embryonic lethality, with a deficiency of Txnrd2 altering cardiac structure [28,29]. Txnrds play a direct role in regulating the redox state of thioredoxins (Txn or Trx), which are important thiol mediators of antioxidant responses. Trxs are small proteins that act as a source of electrons for the reduction of many redox-active protein thiols, including those in the thioredoxin-dependent peroxiredoxin enzymes that reduce hydrogen peroxide (Figure 1b) [30]. Trx1 is found in the cytoplasm, whereas Trx2 is located in the mitochondria. Txnrd1 has also been proposed to have an antioxidant function by directly reducing the sulfenic acid form of tyrosine phosphatase 1B following exposure to hydrogen peroxide; however, this Sec-requiring function may be specific for the tyrosine phosphatase 1B, to date this is the only phosphatase known to be directly reduced by Txnrd1 [31]. Additionally, Txnrd2 regulates the redox-state of mitochondrial glutaredoxin 2 [30]. Txnrds have been found to contribute to endothelial function, modulate inflammatory responses, and influence cardiac outcomes in mouse model systems.

In humans there are 4 methionine sulfoxide proteins (MsrA, MsrB1, MsrB2, MsrB3) that modulate reversible methionine oxidation. Methionine oxidation results in diastereomeric forms, Met-S-sulfoxide, which is reduced by MsrA, and Met-R-sulfoxide, which is reduced by the MsrB enzymes. Only the MsrB1 is a selenoprotein [32,33]. Recent studies have shown that Msr enzymes may regulate protein functions by altering the redox status of specific protein Met residues. As discussed further below, the ability of MsrB1 to reduce particular Met residues in actin has significant effects on macrophage functions that are relevant to antioxidant mechanisms [34].

Other selenoproteins also regulate cellular functions that contribute to cardiovascular disease; however, their direct enzymatic functions are less clear. Selenoprotein P is the only mammalian selenoprotein with more than one Sec residue; in humans, mice, and rats its full-length form has 10 Sec residues. Selenoprotein P is a liver-specific secreted protein that has been shown to have an essential role in delivering Se to various tissues, including brain [35]. The absence of selenoprotein P is not fatal, but it leads to neurological deficits in spatial learning and memory in mice fed a standard diet [36]. Circulating selenoprotein P has been reported to be a good biomarker of selenium status [37], although, as discussed further below, it may not always play a beneficial role in the cardiovascular system.

Less is known about the biological functions of the endoplasmic reticulum (ER) proteins, Selenoprotein K, S, and T. Selenoprotein K appears to regulate Ca^2+^ flux and mediates immune responses [38]. Selenoprotein K as well as Selenoprotein S are components of the ER-associated protein degradation complex that removes unfolded and misfolded proteins from the ER to the cytosol for proteasomal degradation [39]. Selenoprotein T may be involved in regulating the early stages of *N*-glycosylation in the ER and it may play an essential role in mediating ER homeostasis, as its knockdown in mice is lethal [40]. Both Selenoprotein S and Selenoprotein T modulate cellular oxidant status.

## 3. The Role of Selenoproteins in Vascular Tone

Endothelial dysfunction can be characterized, in part, as a loss of normal endothelium-dependent vasorelaxation response to flow or to agonists, such as acetylcholine [41]. Endothelial dysfunction is an early marker of atherosclerosis and promotes thrombosis. Loss of bioavailable nitric oxide (NO) is a hallmark of endothelial dysfunction, but alterations to other endothelial vasodilators, such as prostacyclin and endothelium-derived hyperpolarizing factor, can also contribute to vascular tone [42]. NO protects the vascular wall from damage by preventing platelet activation, inhibiting the proliferation and migration of vascular smooth muscle cells, and preventing pro-inflammatory changes to the endothelium. Oxidative stress can promote endothelial dysfunction by facilitating the loss of NO, by mechanisms such as the formation of peroxynitrite from its reaction with superoxide. In addition, oxidative stress can decrease the activity of the endothelial nitric oxide synthase (eNOS) by other means, such as decreasing the availability of eNOS cofactors. GPx1 has been found to regulate endothelial function, as genetic knockdown of GPx1 promotes endothelial dysfunction, in both heterozygous and homozygous knockout mice [43,44,45]. These findings were directly connected to increased ROS in these mice, as aortic and circulating levels of the isoprostane iPf2a, a marker of oxidant stress, were higher in GPx1-deficient mice, and treatment with L-2-oxothiazolidine-4-carboxylic acid, an antioxidant compound that increases intracellular glutathione pools, restored vasodilation in GPx1-deficient mice and significantly lowered isoprostane levels [43,44]. Similarly, hyperhomocysteinemic mice had a significant decrease in GPx1 expression that correlated with a decrease in endothelium-dependent vasodilation [46]. Overexpression of GPx1 in hyperhomocysteinemic mice restored endothelium-dependent vasodilation, and, in an endothelial culture system, overexpression of GPx1 was found to increase NO production [47]. Aged GPx1 knockout mice had progressively diminished endothelium-dependent vasodilation that remained significantly impaired compared to wild type mice up to 12 months of age, with evidence for increased ROS production and diminished eNOS activation [48]. Consistent with these findings in mice, serum Se and erythrocyte GPx1 activity inversely correlated with endothelium-dependent vasodilation in human hypertensive patients [49]. Other studies in rats have shown that long term Se deficiency decreases GPx activity in the vessel wall, as well as in heart, liver, and kidney, to increase tissue ROS [50].

GPx3 regulates endothelial function, indicating a role for extracellular antioxidant enzymes in maintaining endothelial homeostasis and regulating bioavailable NO. In GPx3 knockout mice, endothelium-dependent vasodilation was decreased as was circulating cGMP, a marker of NO production [22]. Endothelial-specific knockout of GPx4 had no overall effect on vascular homeostasis in mice fed normal diets; however, as discussed further below, decreased expression of GPx4 in combination with other sources of oxidant stress, such as oxidized LDL or dietary restriction of vitamin E, promoted vascular damage and/or ferroptosis [51,52].

Txnrds were also shown to regulate vascular tone. Their inhibition with pharmacological reagents increased hydrogen peroxide production and inhibited soluble guanylyl cyclase to decrease both endothelium-dependent responses (in response to acetylcholine) as well as endothelium-independent vascular relaxation (measured by responses to an NO donor) [53]. It is the NO-mediated activation of soluble guanylyl cyclase in vascular smooth muscle (VSMC) cells that results in cGMP production to mediate vascular relaxation. Endothelial-specific overexpression of Txnrd2 enhanced vascular reactivity by increasing bioavailable NO and decreasing oxidant production [54], whereas mice with endothelial-specific deletion of Txnrd2 (Txnrd2^ECKO^) have reduced flow-mediated dilation and enhanced production of peroxynitrite [55,56], confirming an important role for Txnrd2 in mediating vascular homeostasis.

Although excess ROS, like hydrogen peroxide, can be detrimental and lead to vascular dysfunction, a number of studies have identified hydrogen peroxide as an endothelial-derived hyperpolarizing factor that can mediate endothelium-dependent relaxation in certain circumstances, such as in response to arachidonic acid in cerebral vessels or in response to acetylcholine in other vessels following their exposure to angiotensin converting enzyme inhibitors [57,58]. Excess antioxidant enzymes, such as GPx1 [57] or catalase [58], were able to attenuate these peroxide-mediated responses, illustrating how excess ‘protective’ enzymes can have unexpected deleterious effects by removing necessary oxidants.

## 4. The Role of Selenoproteins in Inflammation and Atherogenesis

**Figure 2:**
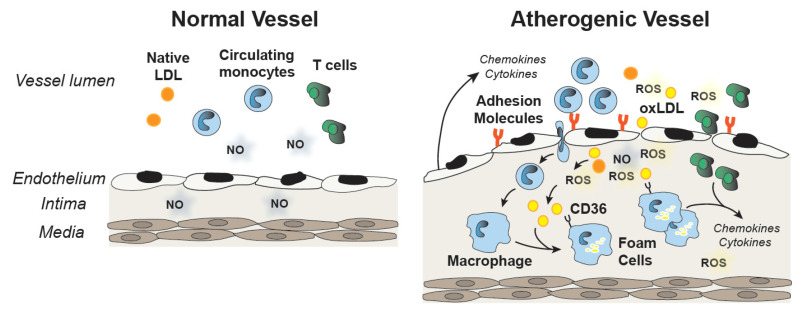
Plaque development in blood vessels. The normal vessel has an endothelial cell layer that produces nitric oxide (NO). Endothelial cell dysfunction and an accumulation of reactive oxygen species (ROS) promotes the pro-inflammatory activation of endothelial cells, including the release of chemokines and cytokines and the expression of adhesion molecules that contribute to the uptake of monocytes and T cells in the intima. Conversion of monocytes to macrophages and foam cells that take up oxidized LDL (oxLDL) through CD36 receptors as well as the activation of T cells causes the release of additional proatherogenic chemokines and cytokines and plaque development.

Endothelial dysfunction and oxidative stress have been implicated in the pathogenesis of atherosclerosis. Decreased NO and increased ROS decrease the barrier function of the endothelium; increase the production of pro-inflammatory cytokines, such as TNF-α; and promote the upregulation of adhesion molecules and chemokines, such as monocyte chemoattractant protein (MCP1). Recruitment and vascular infiltration of leukocytes and monocytes into the vessel wall foster atherogenic changes in the intima. Monocytes differentiate into macrophages and their subsequent uptake of oxidized LDL contributes to the formation of foam cells and fatty streaks in the vessel wall. Cytokine production by macrophages and leukocytes in the artery wall contribute to the development of atherosclerotic lesions, and selenoproteins are involved in many of these steps (Figure 2).

In the context of a high cholesterol diet, Se and vitamin E were found to be important for maintaining endothelium-dependent vasorelaxation in rat [59]. Subsequent studies showed that treatment with Se and vitamin E reduced atherosclerotic lesions in the hypercholesterolemic rabbit to a greater extent than vitamin E alone, with no change in plasma lipids [60]. An earlier study reported a similar protective measure of Se treatment, either alone or in combination with vitamin E, in high fat diet-fed rabbits that correlated with decreased lipids and decreased plasma malondialdehyde, a marker of lipid peroxidation [61]. Taken together, these studies suggest Se may have a protective role in atherosclerosis that is mediated, at least partially, via antioxidant mechanisms; however, selenoproteins were not analyzed in these studies.

In C57Bl/6 mice fed a high fat diet, knockdown of GPx1 did not enhance the development of atherosclerotic lesions, most likely due to several compensatory factors including an attenuation in the expression of monocyte chemoattractant protein-1 [62]. The high fat diet produces only small simple lesions in the aortic sinus of C57Bl/6 mice. Typically, the ApoE knockout mouse or the LDL receptor (Ldlr) knockout mouse, which develop larger, more complex atherosclerotic lesions, are used in experimental studies of atherogenesis [63]. ApoE deficient mice will develop atherosclerosis on a normal chow diet, although a high fat or Western diet is often used to accelerate disease progression. Ldlr deficient mice, by contrast, require a high fat diet to develop significant disease and hyperlipidemia. Compared to wildtype mice, ApoE mice on a normal chow diet have increased aortic expression of many antioxidant enzymes, including GPx1, GPx3, GPx4 and Txnrd prior to the development of atherosclerosis, but during lesion development, the expression of these antioxidant enzymes declines [64]. In the context of the ApoE knockout on a Western diet, knockdown of GPx1 accelerated atherosclerosis in mice as well as in mice with streptozotocin-induced diabetes [65,66], indicating that in susceptible models GPx1 regulates atherogenesis. In these studies, GPx1 deficiency was found to enhance aortic and mitochondrial ROS production [65] and upregulate proinflammatory and profibrotic markers [66]. Consistent with the proatherogenic effects of GPx1 deficiency, ebselen, a GPx-mimetic, was found to attenuate lesion development in a diabetic-ApoE deficient mouse [67].

In human patients with coronary artery disease, red blood cell GPx1 activity was a strong predictor of future cardiovascular (CV) events, with a hazard ratio of 0.29 (95% confidence interval, CI, 0.15–0.58; *p* < 0.001) for the lowest tertile compared to the highest tertile [68]. Moreover, in coronary artery disease patients, the combination of lowest GPx1 activity and highest plasma homocysteine was associated with the greatest risk for future CV events (HR 3.2; 95% CI, 1.8 to 5.6; *p* < 0.0001) [69]. In a case-control study, lower GPx1 activity was associated with coronary artery disease (4.72; 95% CI, 1.61–13.79) and was inversely correlated with severity of disease [70].

Mechanistically, studies in endothelial cells show that GPx1 modulates pro-atherogenic responses to intracellular oxidants generated by cyclic stretch [71]. Similarly, GPx1 attenuates inflammatory responses following cytokine or endotoxin exposure [72,73], whereas GPx1 deficiency alone upregulates pro-inflammatory markers in endothelial cells [72,74]. Overexpression of GPx1 or pretreatment with antioxidants, such as *N*-acetylcysteine, can mitigate inflammatory responses caused by inflammatory agents. Lack of GPx1 activates NFkB and MAPK pathways, increases oxidant stress, promotes monocyte binding to adhesion molecules on endothelial cells, and decreases activation of eNOS [74,75]. These studies illustrate how GPx1 mediates many of the steps in atherogenesis. Thus, GPx1 mitigates oxidative stress to preserve bioavailable NO, thereby attenuating immune responses that contribute to atherosclerotic plaque formation and progression.

GPx4 also mediates the development of atherosclerosis by regulating the oxidation of cellular lipids. Thus, overexpression of GPx4 was reported to decrease atherogenesis in ApoE deficient mice [51]. Specifically, lesion area and the accumulation of F_2_-isoprostanes in the aorta were decreased by overexpression of GPx4. In isolated mouse endothelial cells, overexpression of GPx4 decreased the phospholipid-mediated accumulation of intracellular and extracellular lipid hydroperoxides, decreased the upregulation of adhesion molecules and monocyte binding to cells, and enhanced cell viability. In another study, the effects of ferroptosis on atherogenesis was examined. As found in earlier studies, atherosclerosis in ApoE mice was associated with a decrease in the expression of GPx4 [64] as well as changes in other markers of ferroptosis [76]. Consistent with a role for ferroptosis in the development of atherosclerosis, ferrostatin-1 (Fer-1), a ferroptosis inhibitor that decreases lipid peroxidation, abrogated lesion development and decreased markers of lipid peroxidation in the thoracic aorta similar to statin treatment [76]. In vivo, the protective effect of ferrostatin coincided with increased GPx4 expression and restoration of other ferroptotic markers to their normal levels. Similarly, in cultured mouse endothelial cells exposed to oxidized LDL, GPx4 expression was suppressed and ferroptotic pathways were activated. Furthermore, oxidized LDL promoted inflammatory changes, including an upregulation of adhesion molecules, in mouse endothelial cells. In cultured cells, Fer-1 decreased ferroptosis in response to oxidized LDL, increasing cell viability, upregulating GPx4 expression, and attenuating pro-inflammatory activation. Taken together, these findings shed new light on the role of GPx4 and ferroptosis in atherogenesis.

Selenoprotein S was found to be highly expressed in many tissues in the rat, including the aorta, where its function in both endothelial and vascular smooth muscle cells was found to be protective. A high fat diet, in context of the atherogenic-susceptible Ldlr-deficient mouse, was found to upregulate the expression of Selenoprotein S in lesions, and the cytokine TNF-α was found to increase its expression in human endothelial cells [77]. In endothelial cells, knockdown of Selenoprotein S augmented, whereas overexpression attenuated, the deleterious effects of TNF-α on cell viability and eNOS expression. Similarly, Selenoprotein S deficiency enhanced TNF-α-mediated adhesion molecule expression, monocyte adhesion, and the upregulation of inflammatory cytokines in endothelial cells, whereas its overexpression attenuated these inflammatory changes. Taken together these findings suggest that upregulation of Selenoprotein S in response to inflammation is part of a protective, anti-atherosclerotic response. Although the mechanism is unclear, Selenoprotein S may mediate some of its functions by regulating the accumulation of ROS in endothelial as well as smooth muscle cells [77,78]. In vascular smooth muscle cells, Selenoprotein S was found to mediate oxidative stress and ER-stress responses, as cells lacking selenoprotein S were more sensitive to hydrogen peroxide- and tunicamycin- mediated activation of the ER-stress responses, cell death, and apoptosis [78].

MsrB1 reduces methionine sulfoxide residues in proteins converting them back to methionine. There is a growing body of evidence that oxidation and reduction of specific protein methionine residues modulate biological processes [32]. In LPS-exposed macrophages, MsrB1 mediates the reduction of Met-R-sulfoxide in actin to influence the actin polymerization state and macrophage function [34]. Up-regulation of MsrB1 in response to LPS appears to mediate protective actions, including anti-inflammatory cytokine production in macrophages [79].

Unlike the atheroprotective effects of many other selenoproteins, the ER protein Selenoprotein K may potentiate atherosclerosis by regulating oxidized LDL uptake in macrophages, thereby regulating foam cell formation [80]. Specifically, Selenoprotein K deficiency was found to decrease the palmitoylation of CD36, a scavenger receptor that plays a role in taking up oxidized LDL. Decreased CD36 palmitoylation attenuated cytokine-stimulated accumulation of CD36 on macrophage cell membranes and the clustering of CD36 in membrane lipid rafts. These changes in CD36 membrane localization correlated with a reduced capacity to take up LDL, decreasing foam cell formation. In vivo, transfer of bone marrow from Selenoprotein K knockout mice into high fat diet-fed Ldlr-deficient mice decreased significantly atherosclerotic lesion formation, indicating an important role for Selenoprotein K in promoting key atherogenic processes such as foam cell formation.

In endothelial cell model systems, Txnrd1 was shown to contribute to the activity of NFkB, a transcription factor that mediates the upregulation of many pro-inflammatory genes. Thus, the activity of Txnrd1 regulates the transcriptional activity of NFkB and thereby promotes inflammation [81]. In addition, it has been proposed that Txnrd1 suppresses the activation of Nrf2; inactivation of Txnrd1 with inhibitors or by selenium deficiency leads to the activation of Nrf2 signaling [82]. The transcription factor Nrf2 protects against oxidative stress and electrophilic (reductive) stress by coordinating the transcriptional upregulation of genes involved in the synthesis of glutathione, as well as the genes encoding other antioxidant and redox-regulatory enzymes. These findings suggest that compensatory upregulation of Nrf2 following Txnrd1 inhibition may help to maintain redox balance in cells, in part, by activating GSH-dependent pathways.

## 5. The Role of Selenoproteins in Vascular Remodeling and Angiogenesis

Vascular smooth muscle cell (VSMC) proliferation and migration into the intima can contribute to restenosis or re-narrowing of blood vessels following balloon angioplasty and stenting. Studies in both patients and animal model systems suggest that oxidative stress contributes to neointimal proliferation and remodeling of blood vessels following angioplasty and stenting [83]. Loss of intact endothelium caused by this procedure also contributes to VSMC proliferation and migration, in part, due to the loss of bioavailable NO. GPx1-ApoE double knockout mice were used to study vascular remodeling following balloon angioplasty and stenting (BAS). In the context of ApoE deficiency, GPx1 knockout enhanced VSMC proliferation, promoted medial ROS generation, and inhibited the regeneration of the endothelium following BAS [84]. Vascular injury also upregulated GSH levels and the levels of GSH-synthesizing agents. Mechanistic studies focused on the role of ROS1, an orphan receptor tyrosine kinase that can promote cell growth and survival [85], in vascular remodeling. ROS1 was upregulated in in-stent neointima of patients as well as in aortic lysates from GPx1-deficient ApoE mice following BAS. Silencing of ROS1 significantly decreased proliferation of the GPx1-ApoE-deficient VSMC. Ultimately, the excess activation of ROS1 (measured by its phosphorylation) was attributed to increased glutathiolation of the tyrosine phosphatase, SHP-2, a modification that decreased its ability to deactivate ROS1. These findings highlight the role of GPx1 in protecting against adverse vascular remodeling.

Consistent with the decreased regenerative capacity of endothelial cells in the setting of the BAS model, earlier studies reported that GPx1 deficiency attenuated neovascularization in a hindlimb model of ischemia [86]. The attenuation of revascularization was due, in part, to decreased numbers of endothelial progenitor cells following ischemia and the enhanced sensitivity of GPx1-deficient progenitors to ROS-induced apoptosis [86]. An induced, endothelial-specific Txnrd2 knockout (Txnrd2^ECKO^) also decreased neovascularization following hindlimb ischemia. Endothelial progenitor cells from Txnrd2^ECKO^ mice produced more ROS than wildtype progenitor cells, had lower mitochondrial membrane potential, and had decreased tube formation in an in vitro Matrigel assay, an assay that tests the ability of endothelial cells to form capillary-like structures or tubes in the context of extracellular matrix [56].

Other selenoproteins influence the angiogenic capacity of endothelial cells. Vitamin E deficiency in culture conditions appears to contribute to the impaired angiogenesis of aortic explants from endothelial cell-specific GPx4 knockout (GPx4^ECKO^) mice [52]; in vivo these vessels are normal. Furthermore, deletion of the GPx4 gene caused death in endothelial progenitor cells grown in culture. In vivo, 80% of the GPx4^ECKO^ mice on a vitamin E-deficient diet died. Overall, vitamin E deficiency in the context of GPx4^ECKO^ caused profound histopathological alterations in the endothelium, including separation of endothelial cells from the basement membrane, and endothelial cell death. Overexpression of Selenoprotein P in mice impaired neoangiogenesis in the hindlimb-ischemia model [87], whereas its decreased expression in heterozygous knockout mice had the opposite effect, increasing revascularization in the hindlimb-ischemia model. Mechanistic studies found attenuated responses to VEGF in human endothelial cells exposed to Selenoprotein P. Specifically, VEGF-mediated cell proliferation and tube formation in an in vitro Matrigel assay were diminished by Selenoprotein P. Furthermore, Selenoprotein P attenuated VEGF-mediated ROS production to decrease activation of VEGF-receptor (VEGFR2) signaling. In the angiogenesis studies, exposure of endothelial cells to buthionine sulfoxide (BSO), an inhibitor of glutathione synthesis, was able to attenuate the Selenoprotein P-induced loss of VEGFR2 signaling in endothelial cells, suggesting that Selenoprotein P may be acting by increasing the expression of GSH-requiring selenoenzymes, such as GPx1, to regulate signal transduction [87]. Earlier studies showed that exogenous Selenoprotein P increased GPx1 in endothelial cell cultures to protect cells from excess hydrogen peroxide [88]; however, the effect of Selenoprotein P on the expression of other selenoproteins, such as GPx1, was not examined in this angiogenesis study.

Txnrd also mediates the ability of endothelial cells to produce and respond to VEGF. In endothelial cells isolated from healthy bovine mammary tissue, Se depletion increased the production of VEGF, as well as the cell surface expression of its receptor to stimulate endothelial cell proliferation, migration, and tube formation in a Matrigel assay. This enhanced proliferative response could be mimicked by inhibiting Txnrd activity, whereas knockdown of GPx1 had no effect on endothelial cell growth. The exact mechanism by which Txnrd regulates the increase in VEGF is unknown, but it may be related to cellular redox changes that occur in the absence of Txnrd [89]. As in many other studies performed with Txnrd inhibitors, this study did not clarify whether the conditions inhibited Txnrd1, Txnrd2, or both forms.

## 6. The Role of Selenoproteins in Thrombosis and Stroke

Oxidative stress and lack of bioavailable NO can promote thrombosis, and in ischemic stroke, ROS generation plays a role in cerebral injury following reperfusion. The stroke susceptibility of GPx1-deficient mice was explored using the middle cerebral artery-ischemia reperfusion model (MCA I/R). In these studies, GPx1 deficiency augmented apoptotic responses in neurons [90], increased oxidant stress, and promoted the activation of NFkB [90,91]. GPx1-deficient mice also had a decrease in post-ischemia microvascular perfusion with increased vascular permeability and increased MMP9 expression [92]. Pre-treatment of GPx1-deficient mice with ebselen, a GPx-mimetic, improved microvascular perfusion, decreased MMP9 expression, and decreased vascular permeability, resulting in a decreased infarct size. Consistent with this observation, GPx1 overexpression in mice lessened damage in a stroke model [93]. Taken together, these studies illustrate the importance of redox balance in limiting cerebral damage in an ischemia-reperfusion model of injury. Consistent with these findings, in a human trial, the drug ebselen was used to treat acute ischemic stroke with significant improvement in outcome in patients who started ebselen within 24 h of stroke onset [94].

GPx3 deficiency also has a role in promoting thrombotic disease and enhancing injury in stroke. This association was first discovered in related patients with childhood cerebrovascular thrombotic disease [95,96]. The genetic defect for this condition has not been characterized; however, GPx3 activity was decreased by 50% in these affected patients, and activation of platelets and platelet P-selectin expression could not be blocked by NO administration. These findings suggest that circulating levels of GPx3 can regulate platelet homeostasis and modulate the risk of thrombotic disorders, perhaps via oxidative inactivation of NO and its anti-thrombotic actions. Other studies have shown that GPx3 expression is upregulated in hypoxia, suggesting it may protect against ROS-induced damage during ischemia-reperfusion injury, such as in stroke [97]. In human samples, a variant haplotype (H-2) in the *GPX3* promoter region that was associated with increased risk of arterial ischemic stroke decreased basal and hypoxia-induced expression in a gene reporter assay [98]. Subsequent studies found that this haplotype was a strong, independent risk factor for cerebral venous thrombosis [99]. Another study similarly found an association of the H2-haplotye with arterial ischemic stroke in children but not for thromboembolic or cerebral sinovenous thrombosis in children [100].

Studies in a GPx3 knockout mice confirmed the findings in GPx3-deficient patients [22]. Thus, lack of GPx3 was found to be prothrombotic: deficiency of GPx3 attenuated bleeding times, elevated plasma P-selectin, and increased platelet aggregation to ADP in both in vitro and in vivo assays of platelet function. Furthermore, deficiency of GPx3 decreased circulating cGMP and caused endothelial dysfunction, consistent with a lack of bioavailable NO. In the middle cerebral artery-ischemia reperfusion model (MCA I/R), GPx3 deficiency enhanced cerebral injury, augmenting infarct size and increasing neurological impairment. Platelet activation was shown to account for increased injury in this model as treatment with clopidogrel, a platelet inhibitor, significantly decreased stroke volume and improved neurological function. Oxidants also play a role in mediating injury in the MCA I/R model, as treatment with MnTBAP, an antioxidant, lessened brain damage following MCA I/R in GPx3 knockout mice. Interestingly, subsequent studies in a renal insufficiency model found that dysregulation of NO and cardiac microthrombosis contributed to cardiac dysfunction in GPx3-deficient mice [101], suggesting that lack of GPx3 augmented vascular thrombosis in response to physiological changes that occur in chronic kidney disease. Asymmetrical dimethylarginine (ADMA), an inhibitor of NO, is a known prothrombotic factor that is elevated in patients with renal insufficiency. In the mouse renal insufficiency model, ADMA was elevated to a similar extent in wildtype and GPx3-deficient mice; however, platelets from GPx3-deficient mice were more sensitive to ADMA-induced aggregation than those from wild type mice. Ebselen treatment was found to decrease platelet aggregation and improve cardiac function in renal insufficient GPx3-deficient mice, illustrating the role of excess ROS in the disease phenotype caused by GPx3 deficiency.

Interestingly, Txnrd2^ECKO^ mice developed vascular microthrombi. As in the GPx3-deficient mice, Txnrd2^ECKO^ mice also had endothelial dysfunction [56], confirming a role for NO insufficiency in promoting a prothrombotic environment and indicating that GPx3 and endothelial Txnrd2 are important regulators of bioavailable NO and thrombosis.

As discussed in the previous section, the combination of induced-endothelial-specific GPx4 deficiency with vitamin E deficiency [52] caused endothelial cell death in vivo. Furthermore, the absence of GPx4 and vitamin E was found to result in early death or paralysis due to thrombus formation in many organs. These mice also had elevated systemic blood pressure, increased lipid peroxidation, and severe structural alterations of the endothelium in many vascular beds. These findings highlight the importance of the redox state for endothelial cell homeostasis and as a mechanism that regulates thrombogenesis. These findings also illustrate that vitamin E is sufficient to compensate for GPx4 deficiency in endothelial cells.

## 7. The Role of Selenoproteins in Cardiovascular Development

The *TRSP* gene in humans and *Trsp* gene in mice encode the tRNA^(Ser)Sec^. The tRNA^(Ser)Sec^ is essential for the expression of selenoproteins. Thus, to study the role of selenoproteins in particular cell types, LoxP-Cre techniques have been used [102]. Endothelial-specific knockdown of *Trsp* in mice was embryonically lethal, whereas its knockdown in cardiac and skeletal myocytes caused perinatal lethality with severe myocarditis. Txnrd1-deficiency causes an early embryonic lethality with an overall growth defect, and fibroblast-specific cells from these animals do not proliferate in culture; however, cardiac-knockout of Txnrd1 is not lethal and does not cause a cardiac phenotype [29]. By contrast, Txnrd2 knockout embryos die at 13 days as a result of defects in hematopoiesis and cardiac development [28], and cardiac-specific Txnrd2 knockout results in a fatal dilated cardiomyopathy with structurally abnormal cardiomyocytes. Furthermore, mice with induced knockdown of cardiac Txnrd2 are more susceptible to cardiac injury [103]. Mutations in the human *TXNRD2* gene that affect the flavin-adenine dinucleotide binding domain result in a dilated cardiomyopathy [104].

GPx4 deficiency in mice is embryonically lethal, and in humans, rare premature-truncation mutations of *GPX4* were found to cause Sedaghatian-type spondylometaphyseal dysplasia, an autosomal recessive, neonatal lethal condition that causes developmental defects in cardiac, nervous, and skeletal systems [105].

## 8. The Role of Selenoproteins in Cardioprotection

The importance of Se in cardiac disease can be illustrated by a cardiomyopathy, Keshan disease, that is endemic to regions of China with low Se availability. In these regions, Se supplementation has been shown to decrease the incidence of disease and to increase the plasma, erythrocyte, and platelet levels of glutathione peroxidase (GPx) activity [106]. It has been suggested that a viral determinant, like Coxsackie virus, may influence the occurrence of this cardiomyopathy. Experimental evidence in mice showed that Se deficiency or lack of GPx1 could enhance the mutagenicity of the Coxsackie virus to cause cardiomyopathy [107,108], supporting a role for Se and selenoproteins in regulating the virulence of this infective agent. In a 10-year follow-up study, long-term Se supplementation was associated with improved survival in chronic Keshan disease with congestive heart failure [109]. Taken together these findings implicate Se-deficiency in the pathogenesis and progression of Keshan cardiomyopathy.

In vitro, cardiomyocytes cultured under Se-deficient conditions have increased oxidative stress with an upregulation of several inflammatory genes, and impaired mitochondrial function with significant alterations in basal and maximal respiration [110]. Furthermore, mice fed a Se deficient diet and those fed excess Se both developed cardiac fibrosis with cardiac dysfunction [111], suggesting there is a balance between the beneficial and harmful of effects of Se and selenoproteins.

Cardiac hypertrophy in response to triiodothyronine or isoproterenol treatment was found to upregulate the expression of MsrB1, GPX3, GPx4, and Txnrd1 [112], leading to the speculation that their increased expression may mitigate cardiac damage following induction of hypertrophy. GPx1 was not upregulated by these hypertrophic reagents; however, other studies suggest that GPx1 also plays an important role in mitigating the negative effects of cardiac hypertrophy and cardiac ischemia-reperfusion injury. Thus, in a model of Angiotensin II induced cardiac hypertrophy, absence of GPx1 enhanced left ventricular hypertrophy and decreased cardiac function [113]. Angiotensin II is known to increase the activation of NADPH oxidases to increase superoxide generation, resulting in hypertension, vascular remodeling, and cardiac hypertrophy. Lack of GPx1 in this model would likely enhance the accumulation of ROS to promote pathogenic changes. In another model of cardiac hypertrophy involving exposure to the chemotherapeutic agent doxorubicin, GPx1 deficiency increased cardiac and mitochondrial dysfunction and cardiac apoptosis compared to wildtype mice [114], whereas overexpression of GPx1 was found to preserve contractile and mitochondrial function [115]. Similarly, in mouse models of ischemia-reperfusion injury, hearts from transgenic mice overexpressing GPx1 had improved recovery of cardiac function and decreased infarct size compared to wild type hearts [116]. Interestingly, in GPx1 knockout mice, male hearts were more susceptible to contractile and diastolic dysfunction than female hearts. The male hearts showed increased oxidant stress compared to the female hearts. This difference may be caused, in part, by protective antioxidant mechanisms in the female GPx1 knockout mice that involve ascorbate and nitric oxide metabolism [117]. Another study that focused on the mitochondrial dysfunction following cardiac ischemia-reperfusion injury found that deficiency of GPx1 augmented mitochondrial dysfunction post-reperfusion. Thus, hearts from GPx1-deficient mice had decreased expression of proteins involved in oxidative phosphorylation, and these changes correlated with decreased mitochondrial membrane potential, increased mitochondrial ROS production, increased mitochondrial DNA damage, and decreased mitochondrial respiration following reperfusion [118].

In a Langendorff model of global no-flow ischemia-reperfusion injury in isolated hearts, overexpression of the mitochondrial form of GPx4 improved contractile function compared to that in wildtype hearts [119]. Mitochondrial lipid peroxidation was lower and mitochondrial electron transport function was preserved with GPx4 overexpression. Overexpression of the mitochondrial form of GPx4 similarly improved mitochondrial respiration, decreased ROS, and improved cardiac function in a streptozotocin and hyperglycemia-induced model of diabetes [120]. Taken together, these studies suggest that excess GPx4 mediates cardioprotection by preserving mitochondrial function. Consistent with a role for GPx4 in protecting the heart, deficiency of GPx4, in heterozygous knockout mice, augmented cardiac damage in mice fed a high fat, high sucrose diet. Compared to wildtype mice on the high fat, high sucrose diet, GPx4-deficient mice had increased levels of oxidant stress, cardiac fibrosis, cardiomyocyte hypertrophy, and cardiac mitochondrial dysfunction [121]. Doxorubicin-induced cardiomyopathy correlated with decreased expression of GPx4 and increased lipid peroxidation, leading to ferroptosis and functional impairment of the left ventricular ejection fraction with increased cardiac fibrosis [122]. Consistent with these findings, overexpression of GPx4 attenuated these deleterious changes, whereas heterozygous GPx4-deficient mice had augmented cardiac dysfunction in response to doxorubicin.

The role of Selenoprotein T in cardiac function is not well understood, but a recent study suggests it may promote protection against ischemia-reperfusion injury [123]. In this study, the authors discovered that Selenoprotein T, which is expressed at high levels in embryonic heart and very low levels in the adult heart, is upregulated in isolated rat hearts undergoing ischemia-reperfusion in the Langendorff model system (using 30 min no flow ischemia followed by 120 min reperfusion). To determine whether Selenoprotein T can promote cardioprotection, a peptide encompassing the Selenoprotein T active site was synthesized and introduced into isolated hearts for 20 min at the start of reperfusion. A similar inert peptide was made that lacked the Sec site. Both systolic and diastolic function were improved by infusion of the Selenoprotein T peptide (PSELT), whereas the inert peptide had no significant effect on these parameters following ischemia-reperfusion. Infarct size was also lower in the hearts treated with PSELT. Compared to ischemia-reperfusion, PSELT treatment enhanced the activation of members of the RISK cascade, which is involved in post-ischemic preconditioning, including AKT and ERK-1/2, whereas PSELT suppressed activation of the MAPK-stress response pathway (p38). Furthermore, pro-apoptotic signals such as Bax, cleaved caspase 3, and cytoplasmic cytochrome C were decreased by PSELT treatment. Ischemia-reperfusion-induced increases in nitrotyrosine and ROS were also attenuated by PSELT. Unfortunately, the inert peptide was not used for comparison in the mechanistic studies; nonetheless, these findings are intriguing and suggest a beneficial role of the Selenoprotein T peptide in ischemia-reperfusion injury. Previous studies in other tissues have shown that selenoprotein T regulates oxidative and nitrosative stress, consistent with a beneficial effect of its upregulation during cardiac stress [40].

Txnrdn2 also regulates myocardial protection possibly due, in part, to its role in mediating the release of mitochondrial oxidants and maintaining mitochondrial integrity [124]. Consistent with the protective actions of Txnrdn2, induced, cardiac-specific knockdown of Txnrd2 (Txnrd2^ic^) increased systolic dysfunction and cardiomyocyte death in an ischemia-reperfusion model [103]. Furthermore, mitochondrial structure and function were disrupted by the loss of cardiac Txnrd2. Antioxidant treatment improved cardiac function, restored mitochondrial structure, and decreased cardiomyocyte death, indicating a role for ROS in the dysfunction caused by cardiac ischemia-reperfusion injury. A subsequent study in older Txnrd2^ic^ mice demonstrated that older mice developed significant cardiac dysfunction characterized by decreased fractional shortening and decreased ejection fraction compared to age-matched control mice. Furthermore, ultrastructural analysis of cardiomyocytes from older Txnrd2^ic^ mice showed mitochondrial degeneration and increased lysosomal bodies [125]. As mentioned in the previous section, functional mutations in the human *TXNRD2* gene result in a dilated cardiomyopathy [104]. Interestingly, these mutations show a dominant negative effect on fibroblast cell survival in response to the presence of BSO (which decreases synthesis of cellular GSH thereby inhibiting glutathione peroxidase enzymes). Taken together, these findings suggest an important role for Txnrd2 in maintaining cardiac function.

In contrast with the beneficial role for other selenoproteins in cardioprotection, Selenoprotein P appears to attenuate the activation of endogenous, protective mechanisms that are upregulated following cardiac injury in ischemia-reperfusion [126]. Thus, infarct size, apoptotic TUNEL staining, and caspase 3 activation were reduced in Selenoprotein P knockout mice, and activation of the protective RISK-signaling pathways (Akt, Erk, IGF, S6K) were also enhanced by Selenoprotein P deficiency. Overexpression of human Selenoprotein P in liver (following tail vein injection of constructs) in the context of the Selenoprotein P knockout enhanced infarct size and decreased RISK signaling, suggesting that the presence of Selenoprotein P enhances myocardial ischemia-reperfusion damage. ROS was not measured in this study. It is also not clear whether Selenoprotein P is acting directly or through the regulation of other Selenoproteins to mitigate RISK survival signaling, as the expression of other selenoproteins was not analyzed in this study. Selenoprotein P is an important carrier of Se for the expression of selenoproteins in many tissues [35,88].

In human subjects, plasma levels of Selenoprotein P are considered a good measure of Se status [37]. Selenoprotein P has a higher correlation to serum Se than does serum GPx activity [127]. In a study of cardiovascular disease risk, quintiles of Selenoprotein P concentration were related to the risk of all-cause mortality, cardiovascular mortality, and first cardiovascular event in a Swedish population [128].

## 9. Se and the Risk of Cardiovascular Disease

Many observational studies find an inverse correlation of serum Se to cardiovascular events, a finding that several meta-analysis studies have confirmed. In analyzing 14 prospective observational cohort studies, Flores-Mateo and colleagues [129] reported high selenium vs. low selenium had a pooled relative risk (RR) of 0.85 (95% CI, 0.74–0.99) for acute coronary artery disease. Similarly, Zhang and colleagues [130] reported that the high selenium group had significantly lower risk of cardiovascular disease (CVD) than the lowest selenium group (RR, 0.87; 95% CI, 0.76–0.99) using prospective studies with blood/plasma or erythrocyte Se measurements The association of Se with mortality was also analyzed in another recent meta-analysis by Xiang et al. [131] that solely focused on observational studies. These results found that low circulating Se was associated with a higher risk of all-cause mortality (RR 1.35; 95% CI, 1.18–1.58), and cardiovascular mortality (RR1.35; 95% CI, 1.13–1.63) but did not have a significant association with coronary mortality. Interestingly, subgroup analysis [131,132] suggested that the effects of low Se were more evident in European populations compared to other populations, possibly due to the lower baseline Se levels in the soil and diets in these regions.

In a meta-analysis by Kuria and coworkers [132] that combined observational and randomized controlled trials, there was a non-significant reduced risk of CVD incidence in high Se vs. low Se (RR 0.66, 95% CI, 0.4–1.09) and a significant decrease in CVD morality (RR 0.69; 95% CI, 0.57–0.84) with high Se. Other meta-analyses of randomized control trials alone have found no protective effects of Se supplementation on CVD disease incidence or mortality [130,133,134]; however, in one of these meta-analysis studies, Se supplementation was found to decrease serum C-reactive protein significantly, a marker of inflammation, and increase glutathione peroxidase activity [134]. Similarly, in an observational study of cardiovascular disease in an elderly Italian population [135], low Se correlated with increased circulating CRP levels and upregulation of inflammatory markers in peripheral blood monocytes.

Other studies suggest that Se supplementation may be beneficial when given as part of an antioxidant mix. In analyzing randomized controlled trials in a meta-analysis, Jenkins and colleagues [136] found that Se alone had no effect on the risk of CVD mortality or all-cause mortality, but these parameters decreased when Se was part of an antioxidant mix (RR 0.77; 95% CI, 0.62, 0.98 and RR 0.90; 95% CI, 0.82–0.98). A Swedish trial with supplementation of Se and Q_10_ is an interesting example of using a combination treatment. Se was decreased in the elderly population used in the trial and Q_10_ is known to be decreased with age [137]. In this study, supplements were given for 4 years, and at the end of the treatment Se levels were significantly higher in the treatment group compared to the placebo group. In a long-term analysis 12 years after the intervention, there remained a reduced CV mortality in the treatment group [138].

As mentioned above, given the differences in Se distribution throughout the world [132], there may be less benefit from Se supplementation in some replete areas. Se was proposed to have a U shaped effect on health, with detrimental biological actions arising from low Se and also from high Se [139]. Several studies suggest that excess Se could increase the risk of type 2 diabetes [140,141,142]. Interestingly, in mouse models, both the deficiency of selenoproteins and mice with high levels of Se (resulting in overexpression of some selenoproteins) showed metabolic dysregulation [143]. A previous study reported that GPx1 overexpression can cause insulin resistance, decrease insulin-mediated signal transduction, and cause obesity [11]. Other studies in mice also show similar harmful effects of selenium deficiency and modest selenium supplementation as both conditions promote myocardial fibrosis and cardiac dysfunction [111]. A 10-year follow up of the Denmark PRECISE trial, which treated subjects with various levels of Se supplementation for 5 years, reported that 300 ug Se daily significantly increased all-cause mortality (HR, 1.59; 95% CI, 1.02–2.46), although there was no increased mortality associated with 100 or 200 ug supplementation [144]. Many other possible factors can contribute to the lack of consistent benefits across multiple Se supplementation studies, such as the types of Se supplements, differences in Se sampling, the role of gene polymorphisms, and the complex effects of Se on other metabolic pathways [145,146].

## 10. Discussion

Se has been shown to be an essential micronutrient that modulates cardiovascular functions via its incorporation into selenoproteins as the amino acid selenocysteine. In regions of China with low Se in the soil, decreased dietary intake of Se is linked to Keshan disease, an endemic cardiomyopathy. In observational studies in other populations, low Se has been associated with increased risk of cardiovascular disease. Rare genetic defects that cause GPx3 deficiency result in platelet hyperreactivity and cerebrovascular thrombotic disease, and genetic mutations that encode a dominant-negative Txnrd2 cause cardiomyopathy in patients, indicating the essential functions of these selenoproteins in maintaining cardiovascular homeostasis.

**Figure 3:**
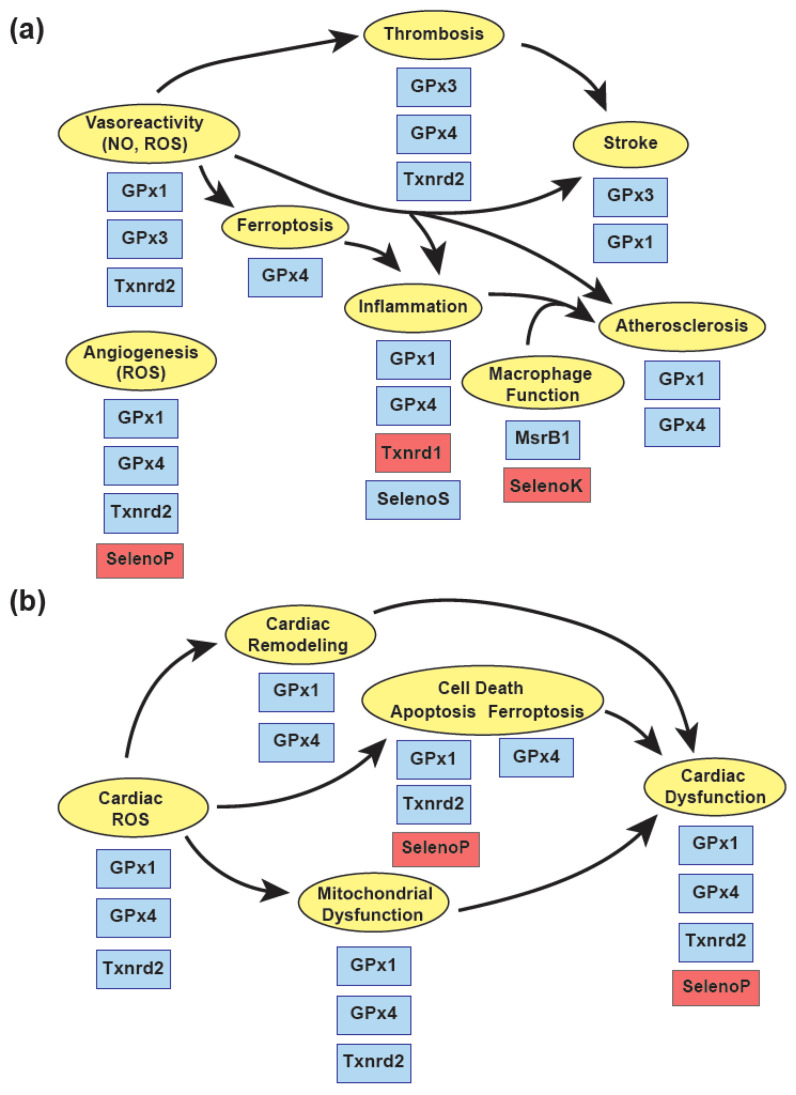
Role of selenoproteins in cardiovascular protection. (**a**) Vascular function. Selenoproteins regulate normal vascular functions, such as vasoreactivity, via their ability to reduce harmful reactive oxygen species (ROS) and preserve bioavailable nitric oxide (NO). Selenoproteins (in blue) also prevent adverse responses such as thrombosis, ferroptosis, inflammation, and thrombosis to prevent diseases such as stroke and atherosclerosis. The actions of Selenoprotein P (SelenoP) and Selenoprotein K (SelenoK) are not protective, as they inhibit angiogenic responses and promote atherogenic macrophage functions, respectively. The actions of Txnrd1 promotes pro-inflammatory signaling via NFkB. Note the effects of GPx4 on vascular NO has not been examined. (**b**) Cardiac function. The selenoproteins (in blue) are protective against cardiac remodeling, cell death, mitochondrial and cardiac dysfunction, except for SelenoP which promotes apoptosis and cardiac dysfunction, as discussed in the text. The effect of SelenoP on cardiac ROS has not been examined.

Experimental studies have shown a role for a subset of redox-active selenoproteins, including GPx1, GPx3, GPx4, and Txnrd2, in protecting against complex cardiovascular diseases, in part, by attenuating the harmful effects of reactive oxygen species (Figure 3). In the vasculature, these proteins have a critical role in maintaining protective endothelial functions that regulate vascular reactivity, prevent adverse vascular remodeling, and allow for essential neoangiogenesis following ischemic injury. Endothelial dysfunction caused by an accumulation of ROS and a loss of bioavailable NO activate VSMC proliferation, enhance inflammatory signaling, and foster a prothrombotic environment promoting atherogenesis, stroke, and cardiac injury. Recent findings on the role of GPx4 in regulating ferroptosis, a type of cell death in response to excess lipid oxidation, suggest that suppression of GPx4 in atherogenesis and in some forms of cardiomyopathy may play a role in disease progression.

Other selenoproteins may regulate protective responses, partially, by decreasing oxidative stress. Thus, by reducing the oxidation of specific protein Met residues, MsrB1 may promote anti-inflammatory responses. Furthermore, Selenoprotein S may have anti-inflammatory effects in endothelial cells and smooth muscle cells, and Selenoprotein T-based peptide treatments may mitigate cardiac dysfunction to decreasing ROS during ischemia-reperfusion.

Unlike the protective effects summarized above, normal expression of some selenoproteins may promote vascular and cardiac dysfunction under disease conditions. Thus, the normal function of Selenoprotein K promotes atherogenesis by fostering foam cell formation. The redox-specific action of Txnrd1 plays an important role in the activation of NFkB, and the antioxidant actions of Selenoprotein P attenuate angiogenesis and contribute to cardiac dysfunction in ischemia-reperfusion, possibly, due to the loss of ROS-mediated activation of signaling pathways. Similarly, overexpression of antioxidant enzymes, such as GPx1, have been shown to reduce cellular ROS to decrease growth factor mediated signaling and promote insulin resistance. These findings suggest the need for additional studies to better understand the role of Se and selenoproteins in cardiovascular health and disease.
